# The Radiology Fellowship Application and Selection Process in the United States: Experiences and Perceptions from Both Sides

**DOI:** 10.1155/2012/875083

**Published:** 2012-07-14

**Authors:** Hyojeong Mulcahy, Felix S. Chew, Michael J. Mulcahy

**Affiliations:** ^1^Department of Radiology, University of Washington, UWMC Roosevelt, P.O. Box 354755, 4245 Roosevelt Way Northeast, Seattle, WA 98105, USA; ^2^Department of Sociology, Central Washington University, Des Moines Teaching Center, Higher Education Center, Building 29, 2400 S 240th Street, P.O. Box 13490, Des Moines, WA 98198, USA

## Abstract

*Objective*. Our purpose was to investigate radiology fellowship directors' and recent fellows' experiences and perceptions with regard to the fellowship application and selection process and to compare these experiences and perceptions. *Materials and Methods*. Institutional review board approval was obtained. We conducted an online survey of the memberships of three radiology subspecialty societies between October 2009 and December 2009 to learn about radiologists' views regarding various aspects of radiology fellowships. *Results*. In the process of selecting fellows, program directors and recent fellows consider performance during the radiology residency and the quality or prestige of the residency program as the most important objective factors, and the personal interview, letters of recommendation, and personality as the most important subjective factors. 25% of the program directors were in the match, and 41% of the recent fellows were in the match. Most (48%) of program directors favored a match, but most (56%) of the recent fellows disfavored participating in a match. Both program directors and recent fellows expressed satisfaction with the fellowship application and selection process. *Conclusion*. There was no majority support for a fellowship match among program directors and recent fellows and less support among recent fellows. Recent fellows appear more satisfied with the current selection and application process than program directors.

## 1. Introduction

The radiology fellowship is one- or two- year clinical training in a subspecialty area that is served after completion of the four-year residency and its prerequisite preliminary transitional internship year. Radiology fellowships therefore represent an optional sixth and seventh years of clinical training. The vast majority of US-trained radiologists complete a fellowship before entering practice. In a survey from 1999, 80% of fourth-year and 84.6% of third-year trainee respondents had accepted fellowship offers or were expected to do so [1]. In a survey from 2009, 93.4% of senior resident respondents planned to pursue fellowships [2]. Fellowship trainees often believe that they are less competitive in the job market without a fellowship, and that they may have an advantage in seeking subsequent employment in the same geographic region as the fellowship. Starting salaries have also been noted to be lower for residency-only graduates [3].

Our purpose was to investigate radiology fellowship directors' and recent fellowship applicants' experiences and perceptions with regard to the fellowship application and selection process and to compare these experiences and perceptions.

## 2. Methods

Institutional review board approval for this study was obtained from the University of Washington Human Subjects Division.

We conducted an online survey of radiologists between October 2009 and December 2009 to learn about radiologists' views regarding various aspects of radiology fellowships. The full survey was comprised of 34 questions, many of which had multiple response items. A subset of questions concerned the fellowship application and selection process and the responses to those questions form the basis for this report. Our survey initially separated the respondents into those who were fellowship program directors (“program directors”), those who had been through the fellowship application process within the past three years (“recent fellows”), and those who were neither. Program directors were asked about their participation in a fellowship match, their most recent complete application cycle, the application requirements for their program, the importance to them of various objective and subjective selection factors, their ability to recruit excellent fellows, their perceptions of the fellowship application and selection process, and whether they favored a fellowship match. There were 55 total items requesting a response from the program directors. Recent fellows were asked about their own application process, their participation in a match, the importance of various objective and subjective selection factors, their perceptions of the process, and whether they favored a fellowship match. There were 43 total items requesting a response from recent fellows. Most items used a 5-point Likert scale for responses; some questions were yes-no, some questions required numeric responses, and most questions offered the opportunity to add unstructured comments.

The survey was made available online using a commercially available survey web site (http://www.surveymonkey.com/; SurveyMonkey.com LLC, Portland, OR, USA). Members of the study population were sent an e-mail describing the survey, its purpose, and the privacy policy applicable to the survey, identifying the investigators, and inviting the recipient to access the survey by following a hyperlink. Survey responses were anonymous.

The study population was drawn from the active memberships of the Association of University Radiologists (AUR), the Association of Program Directors in Radiology (APDR), and the Society for Skeletal Radiology (SSR). The active membership of the AUR is drawn from faculty members of academic radiology departments, mostly radiologists at US medical schools. The active membership of the APDR is drawn from program directors and associate program directors of radiology residency and fellowship programs in the USA The active membership of the SSR is drawn from US-based radiologists whose clinical practice was predominantly musculoskeletal radiology at the time of joining. Three rounds of invitations to participate in a survey regarding radiology fellowships were sent by the societies themselves to each e-mail address in their active membership directories. Approximately, three weeks elapsed between mailings. To account for the three rounds of invitations and the possibility of overlapping memberships, the invitations asked the recipients to take the survey just once. At the time of the survey, there were approximately 750 addresses in the AUR directory, 300 in the APDR directory, and 450 in the SSR directory. Membership in these organizations is not mutually exclusive, but the proportion of overlap was unknown. We also did not know how many program directors or recent fellows were among the recipients of the invitations.

Survey data were analyzed using commercially available statistical software (Stata/MP, Stata Corp LP, College Station, TX, USA). Statistical significance was assessed using Kruskal Walllis tests, which accommodate data tables with cell frequencies of ≤5. Chi-Square probabilities are reported for two-tailed tests; by convention, *P* values ≤ 0.05 indicate a statistically significant association. Open-ended responses and general comments about the survey were collected, categorized, and counted.

## 3. Results

### 3.1. Response Rate

A total of 427 responses to the email invitations were recorded, for an overall response rate of 28.4% (427 of 1,500). There were 201 responses to the AUR mailings, 84 responses to the APDR mailings, and 142 responses to the SSR mailings. However, the actual response rate may have been higher because the 1,500 e-mail addresses that were solicited could have included an unknown fraction of duplicates, invalid addresses, and addresses not in use. It is also unknown whether any radiologists responded multiple times to the invitations; such an action would have decreased the response rate. Among the 427 respondents, there were 114 who identified themselves as program directors, and 37 as recent fellows. This study is thus based on a total of 148 respondents, but not all respondents answered all of the items presented to them.

### 3.2. Demographics

**Figure 1:**
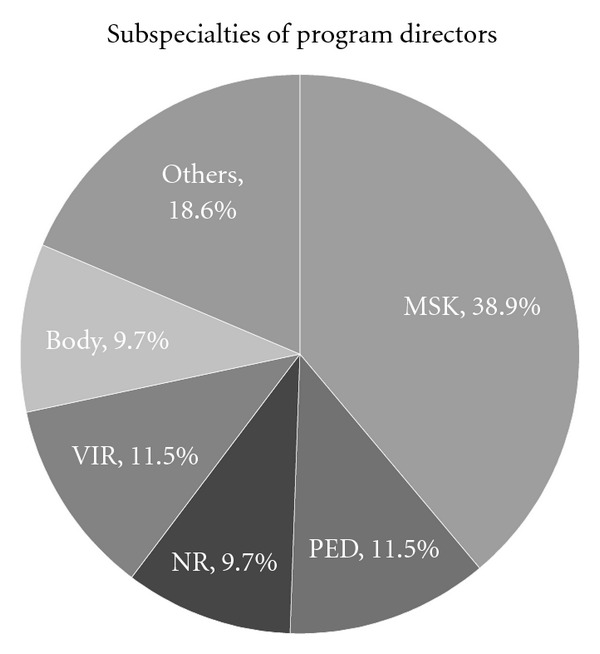
Pie chart showing the subspecialty distribution of program directors.

The subspecialties represented by the program directors were diverse (Figure 1), consisting of musculoskeletal radiology (44), pediatric radiology (13), vascular-interventional radiology (13), neuroradiology (11), abdominal, body, or cross-sectional imaging (11), cardiopulmonary radiology (6), breast imaging (5), nuclear radiology (3), emergency radiology (2), women's imaging (1), MRI (1), and multiple subspecialties (3). There was one incomplete response. Of the multiple subspecialties, it was unclear whether these represented combined multisubspecialty fellowships or directors of multiple separate subspecialty fellowships. The preponderance of musculoskeletal radiology program directors can be explained by the solicitation of their subspecialty society. Indeed, 30 of 44 musculoskeletal radiology program directors responded to the SSR solicitation, while all of the nonmusculoskeletal radiology fellowship directors responded to either the APDR or the AUR solicitations. Of the program directors responding to the question of accreditation, 44% (43 of 97) indicated that their fellowships had ACGME accreditation, and 56% (54 of 97) indicated that they did not. The responding accredited fellowships included pediatric radiology (12), vascular-interventional radiology (10), neuroradiology (10), musculoskeletal radiology (5), nuclear radiology (3), abdominal imaging (2), and multiple (1). As noted below, we looked for significant differences between the responses of accredited and nonaccredited fellowships.

### 3.3. Recent Application Cycle

We asked the program directors about the most recent complete application cycle, including the number of fellowship applications, interviews, offers, and fellows signed. Program directors reported receiving an average of 19 applications, resulting in an average of about 8 interviews, 3 to 4 offers, and between 2 and 3 fellows signed. There were no significant differences between the program directors of accredited and nonaccredited fellowships in this regard. We asked recent fellows about their own application experience. Recent fellows reported submitting an average of 5 to 6 applications, receiving 4 to 5 interview offers, completing 3 to 4 interviews, and receiving 2 to 3 offers. We note that match participants submitted more applications (*P* = 0.046), received more interview offers (*P* = 0.033), and completed more interviews (*P* = 0.005) than those not in the match. Match participants did not differ from nonparticipants in the number of fellowship offers they received. Most (84.6%) recent fellowship applicants felt that they were given adequate time to respond to offers. About 37% (10 of 27) reported that they were internal candidates. Because of the application cycle and completion of the actual fellowship are two to three years apart, the responses of the recent fellows and the program directors did not correspond to the same cycle.

### 3.4. Application Requirements

Nearly all program directors required a curriculum vitae, a personal interview, letters of recommendation, an application form, and a personal statement or equivalent (Table 1). Most program directors also required test scores (e.g., USMLE), and about half required a medical school transcript. A minority required a copy of a medical license or a medical school dean's letter. Only 3% required the payment of an application fee; the amount of these fees was not recorded. While nearly all program directors required a personal interview (97%, 87 of 90) only a third called to check applicants' references (34%, 31 of 90). We surmise that program directors who accepted applicants without requirements had internal candidates who were well known to them.

### 3.5. Selection Factors

**Figure 2:**
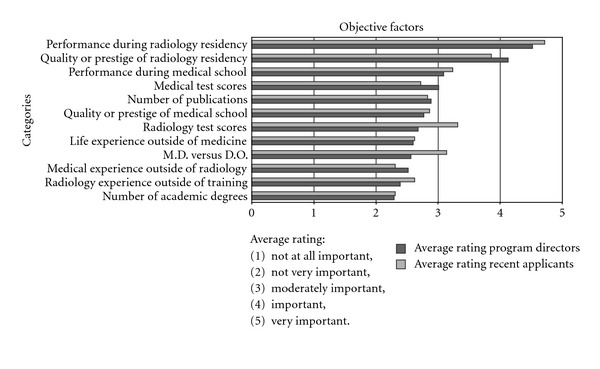
Bar chart comparing the importance of various objective fellowship program selection factors between program directors and recent fellows.

**Figure 3:**
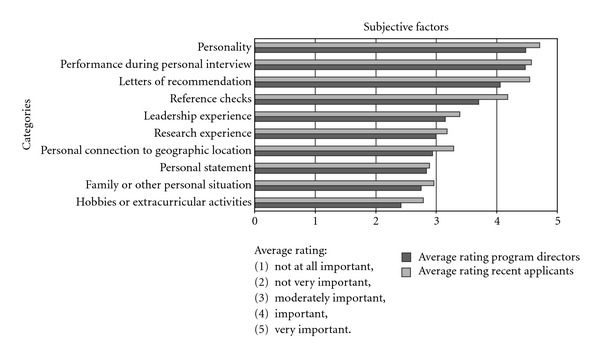
Bar chart comparing the importance of various subjective fellowship program selection factors between program directors and recent fellows.

We asked program directors about the importance of twelve objective and ten subjective factors in the process of selecting fellows. Objective factors were those for which there is a reference standard outside of the program director, even though that standard might itself be subjective. For example, performance during the residency may reflect an aggregate of subjective faculty evaluations and performance observations, but these evaluations and observations are not made by the program director receiving the application. Subjective factors were those judgments that were made by the program director or other faculty involved in the selection process. Among twelve objective factors, only one—performance during the radiology residency—was viewed as “very important” by a majority of the program directors (62%, 54 of 87) responding to this question (Figure 2). A majority of program directors also viewed quality or prestige of radiology residency, performance during the medical school (e.g., rank, grades, AOA), and medical test scores (e.g., USMLE), as moderately to very important factors. Overall, program directors attributed somewhat more importance to subjective factors in the fellow selection process (Figure 3). For three of the ten subjective factors that we asked about (recommendation letters, performance during the interview, and personality), “very important” was the modal response category and it was the majority category for the latter two factors (65.2% and 59.1%, resp.). For two other subjective factors (leadership experience and reference checks), “important” was the modal category. Program directors of accredited fellowships tended to place greater importance on letters of recommendation (*P* = 0.056) and on reference checks (*P* = 0.136) than those of nonaccredited fellowships.

We asked recent fellows about the importance of the same objective and subjective selection factors that we asked program directors about (Figures 2-3). Among objective factors, most recent fellows considered performance during the radiology residency as very important (79%, 23 of 29). Prestige of the residency program was thought to be very important by almost one-third (30%, 8 of 29). Among the other factors, the modal response was either the middle category (“moderately important”) for medical school quality, medical school performance, radiology residency tests, and publications or “not very important” for USMLE scores, medical experience, radiology experience, life experience, other academic degrees, and the M.D. versus D.O. degree. Most recent fellows considered as very important the subjective factors of personality (71%, 20 of 28) letters of recommendation (61%, 17 of 28) and interview performance (61%, 17 of 28). Half of them viewed reference checks as very important (50%, 14 of 28). Views were more divided on the importance of geographic connections and research interests. The middle category was the modal response (“moderately important”) on the remaining subjective factors (personal statement, personal leadership experience, family considerations), with the exception of hobbies, which (54%, 15 of 28) considered not very important.

Recent fellowship applicants tend to place somewhat greater importance on test scores from the radiology residency (in-service exams, ABR) (*P* = 0.008), and directors place less value on the M.D.-D.O. distinction (*P* = 0.051). Both groups consider personality and performance during the personal interview as the two most important subjective factors. Interestingly, program directors place less importance than recent fellows on letters of recommendation (*P* = 0.021) and the results of reference checks (*P* = 0.035).

### 3.6. Ability to Recruit

**Figure 4:**
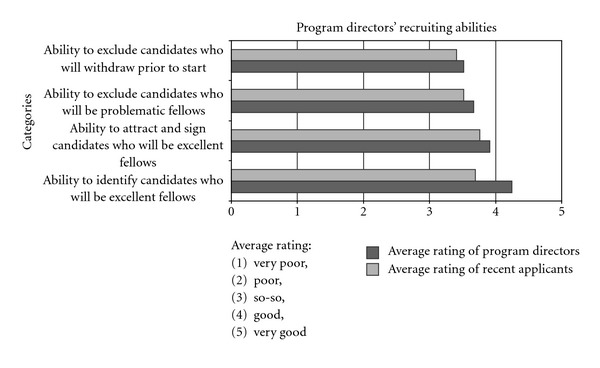
Bar chart comparing program directors' self-assessment of their recruiting abilities with recent fellows' assessments of the program directors' abilities.

We asked program directors about their ability to recruit and sign excellent fellows, and to identify nonviable candidates. Overall, program directors appeared optimistic about their ability to recruit excellent fellows and to weed out nonviable candidates (Figure 4). An overwhelming majority rate their ability to identify candidates who will make excellent fellows as very good or good (83%, 74 of 89), and a strong majority also rate their ability to attract and sign promising candidates positively, that is, either as good or very good (67%, 60 of 89). Directors were slightly less sanguine about their ability to exclude candidates who would be problematic fellows or would likely withdraw prior to start of the fellowship, but even in the latter two areas, about half (56%, 50 of 89, and 51%, 45 of 89, resp.) rated their abilities as either very good or good.

Recent fellows generally had confidence in program directors' recruitment capacities (Figure 4), but perhaps not as much as the program directors had in themselves. The modal response category was “good” for all four of the questions about directors' recruitment abilities. For each question, slightly more than half rated program directors' recruitment abilities as either “good” or “very good.”

### 3.7. Match Participation

Only a quarter (25%, 23 of 89) of program directors responding to our question reported that their program participated in the match during the last complete application cycle. Of the program directors who responded to questions about accreditation and match participation, 50% (20 of 40) of accredited programs participated in the match, while 50% (20 of 40) did not. Neuroradiology and vascular-interventional radiology programs were in the match; pediatric radiology, musculoskeletal radiology, nuclear medicine, and abdominal imaging programs were not. There were 46 non-accredited programs not in the match, and surprisingly, there were two nonaccredited programs (cardiothoracic radiology, body imaging) said to be in the match.

Of the recent fellows who responded to our questions about match participation, most (59%, 16 of 27) did not go through a match, and of the 11 who did, 7 did so for all of their applications, whereas 4 only went through a match for some of their applications.

**Figure 5:**
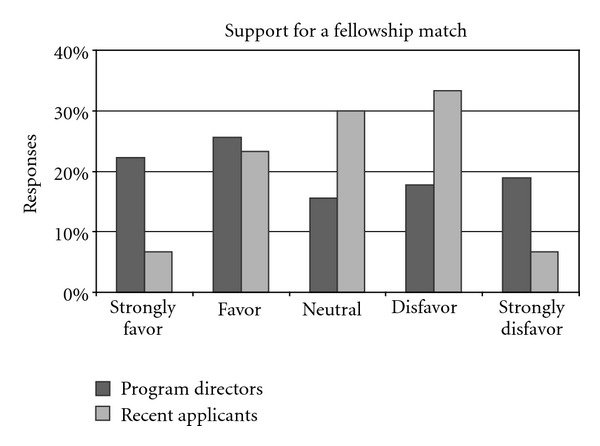
Column chart comparing support for a fellowship match between program directors and recent fellows.

Fewer than half of the program directors were in favor of a match (48%, 43 of 90), while 16% (14 of 90) were neutral, and 37% (33 of 90) disfavored a match (Figure 5). There were 18 unstructured comments regarding match participation. Of these, 10 out of 18 (56%) disfavored participating in a match, 6 out of 18 (33.3%) favored participating in a match, and 2 out of 18 (11%) were neutral. All of the negative comments mentioned bad prior experience as a reason (“Tried but failed”). Positive comments mentioned that it is beneficial and fair to applicants, and it provides a guideline for the process.

Recent fellows were less in favor of a match than the program directors. Whereas 30% (9 of 30) were neutral on this point, 40% (12 of 30) of recent fellow respondents disfavored or strongly disfavored participation, and only 30% (9 of 30) favored or strongly favored it (Figure 5). There were six comments about this question, three favorable and three unfavorable. The unfavorable comments mentioned bureaucracy associated with a match, and an insufficient volume of applicants.

There is a significant association between participation in the match and favorable views on match participation for both directors (*P* = 0.005) and recent fellows (*P* = 0.027). We did not find a significant association between match participation and the importance placed on selection factors on the part of either program directors or recent fellows.

We found no differences between internal candidates and other fellows' support for participation in match. Match participants responded more favorably than other recent fellows to the question regarding participation in match (*P* = 0.027).

### 3.8. Perceptions of the Process

**Figure 6:**
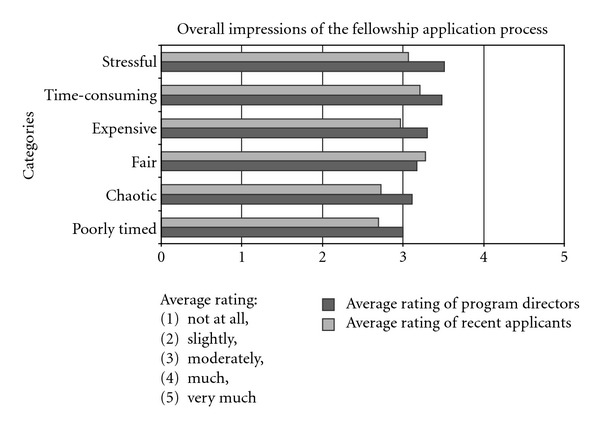
Bar chart comparing how program directors believe applicants regard the fellowship application and selection process with how recent fellows regard the process.

We asked program directors the extent to which they thought fellows viewed the following characterizations of the fellowship application and selection process: chaotic, expensive, fair, time-consuming, stressful and poorly-timed. In all six dimensions, directors' modal response was to attribute the most neutral view to fellows, on a scale ranging from “very much” to “not at all.” There was, however, substantial variation in program directors' responses (Figure 6). Only 30% (27 of 90) of the program directors thought the applicants found the process slightly or not at all chaotic, 24% (22 of 90) slightly or not at all expensive, 11% (10 of 90) slightly or not at all time consuming, 9% (8 of 90) slightly or not at all stressful, and 38% (34 of 90) slightly or not at all poorly timed. Program directors of accredited fellowships were less likely than other directors to believe that fellows experience the recruitment process as chaotic (*P* = 0.006), stressful (*P* = 0.041), or poorly-timed (*P* = 0.005). With regard to fairness, 81% (73 of 90) of the program directors thought the applicants found the process moderately fair to very much fair. Directors participating in the match did not differ significantly from other directors regarding the predictions of fellows' assessments of the fellow selection procedure. There were 8 unstructured comments regarding the question about program directors' perceptions about fellowship applicants' views of the application process. 7 out of 8 (87.5%) were negative views of the process, and most of them (6 of 7) were concerns about timing (“it is too early in many cases”).

We asked recent fellows about the extent to which they did or did not view the selection process as chaotic, expensive, fair, time-consuming, stressful, and poorly timed. As with the program directors, there was a wide range of responses (Figure 6). Only 40% (12 of 30) of the recent fellows found the process slightly or not at all chaotic, 34% (10 of 29) slightly or not at all expensive, 28% (8 of 29) slightly or not at all time-consuming, 27% (8 of 30) slightly or not at all stressful, and 50% (15 of 30) slightly or not at all poorly timed. With regard to fairness, 83% (24 of 29) of recent fellows found it moderately fair to very much fair. Internal candidates were less likely to view the process as expensive (*P* = 0.005) or time consuming (*P* = 0.032), and more likely to view it as fair (*P* = 0.034). Those with recent match experience were more likely to view the process as expensive (*P* = 0.024), time consuming (*P* = 0.010), stressful (*P* = 0.029), and less fair (*P* = 0.072).

Comparing the program directors' perceptions with the recent fellows' responses, the program directors consistently thought that applicants would find the process worse and less fair than they actually did.

### 3.9. Satisfaction

**Figure 7:**
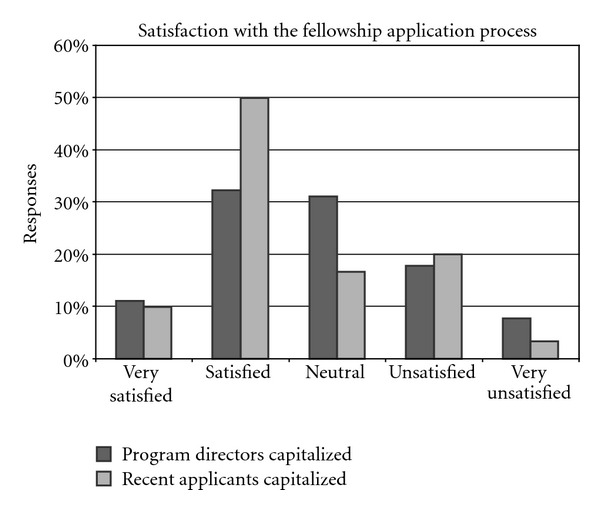
Column chart comparing satisfaction with the fellowship application and selection process between program directors and recent fellows.

The program directors reported overall levels of satisfaction with the fellowship application and selection process that bordered on the satisfied to neutral (Figure 7). More program directors reported being very satisfied (11%, 10 of 90) or satisfied (32%, 29 of 90) with the process than being unsatisfied (18%, 16 of 90) or very unsatisfied (8%, 7 of 90), while 31% (28 of 90) were neutral. Directors of accredited programs expressed higher levels of satisfaction with the fellowship selection process than directors of nonaccredited programs (*P* = 0.058). We did not find a significant difference between program directors who were participants and nonparticipants in the match.

A majority of recent fellows reported being satisfied (50%, 15 of 30) or very satisfied (10%, 3 of 30), whereas fewer reported being either unsatisfied (20%, 6 of 30) or very unsatisfied (3%, 1 of 30) with the process (Figure 7); neutral response was recorded for the remainder (17%, 5 of 30). We found no differences between internal candidates and other fellows on level of satisfaction with the selection process, nor did we find a significant difference between participants and nonparticipants in the match.

### 3.10. What to Change

In an open-ended question, program directors and recent fellows what they would change about the fellowship application and selection process, if they could change just one thing. From the program directors, we received 59 comments: 15 in favor of a match, 12 in favor of a standard timetable, and 11 in favor of moving the process to later in residency (end of PGY-4 or early PGY 5), 7 about more transparency between programs and applicants, 6 about enforcing the commitment of accepted candidates, 3 about attracting better or more candidates, 1 about getting rid of the match, and 4 had no suggestions. From the recent fellows, we received 13 responses: five in favor of a match, two each against a match, against any changes, and about increasing transparency, and single comments about decreasing the cost and delaying the timeline.

## 4. Discussion

Limitations to our study are principally related to sampling. We chose to use e-mail as the method of solicitation because of its negligible cost, and a web-based survey for similar reasons. The AUR, APDR, and SSR were chosen because the authors had access to their membership directories, and as a result, the sample was skewed towards program directors of musculoskeletal fellowships. Musculoskeletal radiology fellowships are mostly nonaccredited and none are in the match. The proportion of recent fellows among the memberships of these societies is low and appears to be reflected in the small size of our sample. Also, responses of recent fellows may have been affected by their postfellowship experience such as job market. We have no basis for speculating how these limitations may have affected our results.

Our survey demonstrates that program directors and recent fellows clearly consider performance during the radiology residency and the quality or prestige of the residency program as the two most important objective factors in the process of selecting fellows. The personal interview, letters of recommendation, and personality appeared to be the most important subjective factors for both program directors and recent fellows. Overall, program directors are fairly optimistic about their ability to recruit excellent fellows and weed out nonviable candidates, and recent fellows generally have confidence in directors' recruitment capacities, although not as much as the directors have in themselves. Interestingly, with regard to the perceptions about the application process, program directors generally seem to think that fellows had a dimmer view of the process than they actually did. Both program directors and recent fellows expressed satisfaction with the fellowship application and selection process, with recent fellows being more satisfied than program directors.

In our survey, program directors and recent fellowship applicants had divided opinions about match participation. Among program directors and recent fellows, there appears to be no majority support for a match, and there was less support among recent fellows than among program directors. The NRMP Radiology Fellowship Match was instituted to address a number of perceived problems with the fellowship application process, and the process was developed through discussion among leaders in radiology, including the National Intersociety Fellowship Application Task Force [4]. However, in the past 6 years, in the absence of any agreement regarding the date for the scheduling of interviews, the timing of the decision process has been pushed back earlier and earlier within the residency, obligating trainees to interview in many subspecialties at the beginning of their third year [2] and make their decision as to subspecialty during their second year. A recently conducted survey of senior residents showed surprisingly negative perceptions of the NRMP fellowship match process, including suggestions of inconsistent participation and rule violations [5]. This is in contrast to previous surveys of fellowship directors, which documented an overall positive response to the match process [6, 7]. Vascular-interventional radiology and neuroradiology fellowships continue to have a match, whereas all other radiology fellowships do not [8].

## 5. Conclusion

There was no majority support for a fellowship match among program directors and recent fellows and less support among recent fellows. Recent fellows appear more satisfied with the current selection and application process than program directors.

## Figures and Tables

**Table 1:** Program directors' responses about application requirements.

Which of the following are required of applicants before a position in your fellowship is offered?		
Answer options	Response percent	Response count
Personal interview	96.7%	87
Curriculum vitae	96.7%	87
Letters of recommendation	95.6%	86
Application form	87.8%	79
Personal statement or equivalent	86.7%	78
Test scores (e.g., USMLE)	63.3%	57
Medical school transcript	52.2%	47
Medical license	37.8%	34
Medical school dean's letter	35.6%	32
Phone call to check references	34.4%	31
Other	14.4%	13
Application fee	3.3%	3
*N* = 90		
